# Supplementary material for: Green Synthesis of Zinc Oxide Nanoparticles Using Aqueous Extract of Pavonia zeylanica to Mediate Photocatalytic Degradation of Methylene Blue: Studies on Reaction Kinetics, Reusability and Mineralization
Source: Int J Mol Sci. 2025 May 15;26(10):4739. doi: 10.3390/ijms26104739 (PMC12112004; doi:10.3390/ijms26104739)
Supplement: Supplementary file 1 [file ijms-26-04739-s001.zip › ijms-3606993-supplementary.pdf]

Table S1. Estimated crystallite size of biosynthesized ZnO-NPs from the aqueous leaf extract of *P. zeylanica*.

	**2θ (deg.)**	**(*hkl*)**	**FWHM**	**Crystallite Size (nm)**	**Average Crystallite Size (nm)**
Phyto-fabricated Pz-ZnO-NPs	31.68	100	0.5391	**16.01**	**19.58**
	34.36	002	0.6865	**12.66**	
	36.22	101	0.5585	**15.64**	
	47.51	102	0.4083	**22.22**	
	56.54	110	0.6837	**13.79**	
	62.78	103	0.5688	**17.10**	
	66.26	200	0.4155	**23.86**	
	67.84	112	0.3958	**25.28**	
	69.02	201	0.3403	**29.61**	
	77.88	202	0.3223	**33.12**	

Table S2. Photocatalytic dye degradation ability of biosynthesized ZnO-NPs from *P. zeylanica*.

**Sl. No.**	**Sample Dosage (g/L)**	**Photocatalytic degradation (%)**
1	0.00	5.26 ± 0.19^{d}
2	0.30	58.23 ± 0.52^{c}
3	0.60	62.82 ± 1.12^{b}
4	0.90	86.68 ± 0.54^{a}
5	1.20	89.32 ± 0.64^{a}

Values are means of three independent replicates (n = 3) and ± indicates standard error. Means followed by the same letter(s) within the same column are not significantly ($p \leq 0.05$) different according to Tukey's HSD.

Table S3. Reusability properties of biosynthesized ZnO-NPs from *P. zeylanica* for Photocatalytic dye degradation.

Reusability	Photocatalytic degradation (%)
1st Cycle	89.32 ± 0.44^{a}
2nd Cycle	88.60 ± 0.82^{a}
3rd Cycle	84.34 ± 0.52^{b}
4th Cycle	83.59 ± 0.46^{bc}
5th Cycle	82.82 ± 0.56^{bc}

Values are means of three independent replicates (n = 3) and ± indicates standard error. Means followed by the same letter(s) within the same column are not significantly ($p \leq 0.05$) different according to Tukey's HSD.

Table S4. Mineralization properties of biosynthesized ZnO-NPs from *P. zeylanica*.

Reaction Time (min)	COD (mg L^{-1})	TOC (mg L^{-1} C)
0	91.26 ± 1.04^{a}	94.12 ± 2.04^{a}
30	78.58 ± 0.76^{b}	82.58 ± 0.56^{b}
60	52.12 ± 0.72^{c}	60.23 ± 0.52^{c}
90	29.53 ± 0.40^{d}	36.53 ± 0.70^{d}
120	18.92 ± 0.42^{e}	25.42 ± 0.42^{e}

Values are means of three independent replicates (n = 3) and ± indicates standard error. Means followed by the same letter(s) within the same column are not significantly ($p \leq 0.05$) different according to Tukey's HSD.
